# Determination of the Critical Value of Material Damage in a Cross Wedge Rolling Test

**DOI:** 10.3390/ma14071586

**Published:** 2021-03-24

**Authors:** Zbigniew Pater, Andrzej Gontarz, Janusz Tomczak, Tomasz Bulzak, Łukasz Wójcik

**Affiliations:** Faculty of Mechanical Engineering, Lublin University of Technology, 36 Nadbystrzycka Str., 20-618 Lublin, Poland; a.gontarz@pollub.pl (A.G.); j.tomczak@pollub.pl (J.T.); t.bulzak@pollub.pl (T.B.); l.wojcik@pollub.pl (Ł.W.)

**Keywords:** damage, cross wedge rolling, calibration test, FEM, experiment

## Abstract

This study investigates the problem of material fracture in cross wedge rolling (CWR). It was found that this problem could be analysed by means of well-known phenomenological criteria of fracture that are implemented in commercial FEM (Finite Element Method) simulation programs for forming processes. The accuracy of predicting material fracture depends on the critical damage value that is determined by calibration tests in which the modelled and real stresses must be in good agreement. To improve this accuracy, a new calibration test is proposed. The test is based on the CWR process. Owing to the shape of the tools and test piece used in CWR, the forming conditions in this process deteriorate with the distance from the centre of the test piece, which at a certain moment leads to fracture initiation. Knowing the location of axial crack initiation in the specimen, it is possible to determine the critical value of material damage via numerical simulation. The new calibration test is used to determine the critical damage of 42CrMo4 steel subjected to forming in the temperature range of 900–1100 °C. In addition, 12 criteria of ductile fracture are employed in the study. The results show that the critical damage significantly increases with the temperature.

## 1. Introduction

Cross wedge rolling (CWR) is a modern technique for producing stepped axles and shafts as well as preforms for press forging. This method has become increasingly popular in recent years, which is undoubtedly connected with the fact that new solutions have been found to overcome its previous limitations. This, in turn, is largely due to the development of both computational methods (software) and computer hardware that make it possible to perform more and more complex analyses within an acceptable time [1].

Generally, the stability of the CWR process can be disturbed by the occurrence of uncontrolled slip, workpiece necking (rupture) and material fracture [2]. The first two failure modes are relatively easy to simulate numerically, which means that their solutions can be produced already at the design stage. The prediction of material fracture poses, however, far more problems. Therefore, many research centres are conducting research aimed at developing effective methods for modelling material fracture in CWR processes.

Studies conducted by Li et al. [3], Yang et al. [4] and Zhou et al. [5] showed that the CWR process is susceptible to ductile fracture. The fracture mechanism is associated with the nucleation of micro voids (usually located close to non-metallic inclusions) and their growth and coalescence into macro voids (due to the effect of both tensile and shear stresses). Other studies focused on the effects of basic parameters of CWR on the formation of internal cracks. Kache et al. [6] found that material fracture is induced by a decrease in the forming temperature. Zhou et al. [7] demonstrated that the growth of cracks results from the use of wedge tools with smaller forming angles but higher spreading angles and thus higher cross-sectional reductions. Zhao et al. [8] also reported that a higher wedge tip fillet accelerates the initiation of material fracture.

The first studies devoted to modelling material fracture in the CWR process were undertaken several years ago. In 2004, Li and Lovell [9] used ANSYS/LS-DYNA to analyse three cases of this rolling process, focusing on the determination of mean stress, stress intensity and effective strain. These researchers also established that the best way to predict fracture is by analysing effective strains. In the following years, the classical ductile fracture criteria implemented as subroutines to commercial computer programs began to be employed on a much wider basis. The first such analyses were performed by Piedrahita et al. [10], who used Forge^®^ and the normalised Cockcroft–Latham criterion to determine the effect of basic parameters of CWR on the initiation of internal cracks. Using the same program and ductile fracture criterion, Silva et al. [11] modelled material fracture by the killing element technique, under which the elements are deleted when the critical damage is reached (its value being determined by standard tensile testing). In 2012, Jia et al. [12] used Deform-3D and a porous material model to determine the effect of basic parameters of the CWR process on the initiation of fracture in 7075 aluminium alloy specimens. Cakircali et al. [13] investigated the fracture of Ti6Al4V alloy using the Johnson and Cook criterion and LS-DYNA program. The critical damage was determined by tensile testing. Zhou et al. [14] used Deform-3D to investigate the multi-wedge cross rolling process, finding that the use of the Cockcroft–Latham criterion to model fracture does not produce sufficiently good results. Novella et al. [15] modified the Oyane–Sato fracture criterion to predict material fracture in the CWR process, using Forge 2011. The criterion was calibrated by hot tensile testing.

The effectiveness of modelling material fracture significantly depends on the calibration method used to determine the critical values of material damage. It is known that the stress state in the calibration test must be in high agreement with the real stress state. This means that the modelling of CWR processes by uniaxial tensile testing does not produce good results. Hence, new calibration tests have started to be developed. Komischke et al. [16] used a two-roll cross rolling process to this end. However, their experiments were limited to the cold forming of steel. Other tests were developed by Pater et al. and they were based on the rotary compression of either a disc-shaped specimen in tool cavity [17] or a cylindrical specimen between two flat plates [18]. Conducted under hot forming conditions, the tests showed that the critical damage depends greatly on temperature. Using the developed calibration tests, Pater et al. [19] evaluated fracture criteria in terms of their suitability for modelling CWR processes. It was found that the criteria which take into account, at least indirectly, the effect of shear stresses yield better results. Similar observations were made by Zhou et al. [20]. In-depth analyses of the stress state in the rotary compression tests and CWR [21] revealed that the stresses are not identical, which may affect the accuracy of modelling material fracture in CWR.

In light of the above, a novel test was developed, in which the stress state is the same as that in cross wedge rolling. An advantage of this test is that it can be performed on rolling mills installed at production plants, in other words—under real, not laboratory, conditions. Hence, the critical damage calculated thereby will make it possible to accurately model material fracture in CWR, using the widely used phenomenological fracture criteria. In this study, the proposed calibration test and 12 well-known fracture criteria are used to determine the critical damage of 42CrMo4 steel subjected to hot forming.

## 2. Fracture Criteria Employed in This Study

This study uses phenomenological criteria of ductile fracture that are widely applied in practice due to their simplicity. They are implemented as subroutines in commercial software dedicated to analysis of forming processes. These criteria are based on the assumption that material fracture is caused by an energy change due to the accumulation of plastic strains, this fracture being described by the following equation (known as the damage criterion):

(1) $$f_{i}=\int_{0}^{\varepsilon_{f}}\Phi \left(\sigma \right)d\varepsilon ,$$

where *f_i_* is the damage function according to the *i*-th criterion, Φ(*σ*) is the function describing the relationship between stress and void nucleation, growth and coalescence, and *ε_f_* is the failure strain.

To predict material fracture, one must know the critical damage value *C_i_*, which is expressed as the value of the function *f_i_* at fracture. Knowing the values of *f_i_* and *C_i_* it is possible to estimate the damage index *w_i_* that describes the percentage probability of fracture initiation:

(2) $$w_{i}=100\%\frac{f_{i}}{C_{i}},$$

with fracture initiating when $w_{i}\geq 100\%$.

Many ductile fracture criteria have been developed over the last several years. Twelve such criteria have been selected for the purpose of this study, depending on their suitability for the analysis of CWR processes. The criteria employed in this study are given below, with the names of their authors and mathematical notations:

- Freudenthal [22]
  (3) $$f_{FREUD}=\int_{0}^{\varepsilon }\sigma_{i}d\varepsilon ,$$
- Cockcroft and Latham [23]
  (4) $$f_{CL}=\int_{0}^{\varepsilon }\sigma_{1}d\varepsilon ,$$
- Rice and Tracey [24]
  (5) $$f_{RT}=\int_{0}^{\varepsilon }\exp\left(\frac{3}{2}\eta \right)d\varepsilon ,$$
- Brozzo et al. [25]
  (6) $$f_{BROZZ}=\int_{0}^{\varepsilon }\frac{2\sigma_{1}}{3\left(\sigma_{i}-\sigma_{m}\right)}d\varepsilon ,$$
- Oyane [26]
  (7) $$f_{OYANE}=\int_{0}^{\varepsilon }\left(1+A\eta \right)d\varepsilon ,$$
- Argon et al. [27]
  (8) $$f_{ARGON}=\int_{0}^{\varepsilon }\left(\sigma_{m}+\sigma_{i}\right)d\varepsilon ,$$
- Oh et al. [28]
  (9) $$f_{OH}=\int_{0}^{\varepsilon }\frac{\sigma_{1}}{\sigma_{i}}d\varepsilon ,$$
- Ayada et al. [29]
  (10) $$f_{AYADA}=\int_{0}^{\varepsilon }\eta d\varepsilon ,$$
- Ko et al. [30]
  (11) $$f_{KO}=\int_{0}^{\varepsilon }\frac{\sigma_{1}}{\sigma_{i}}\left(\langle 1+3\eta \rangle \right)d\varepsilon ,$$
- Zhan et al. [31]
  (12) $$f_{ZHAN}=\int_{0}^{\varepsilon }\left(\sigma_{i}-\sigma_{m}\right)d\varepsilon ,$$
- Lou et al. [32]
  (13) $$f_{LOU}=\int_{0}^{\varepsilon }{\left(\frac{\tau_{max}}{\sigma_{i}}\right)}^{c_{1}}{\left(\frac{\langle 1+3\eta \rangle }{2}\right)}^{c_{2}}d\varepsilon ,$$
- Pater et al. [33]
  (14) $$f_{PATER}=\int_{0}^{\varepsilon_{f}}\left[\left(1-\Phi \right)\frac{\sqrt{3}}{2}\frac{\sigma_{1}-\sigma_{3}}{\sigma_{i}}+\Phi \frac{\sigma_{1}}{\sigma_{i}}\right]d\varepsilon ,$$

where

(15) $$\left\{\begin{array}{c} \Phi =0\;\;\;for\;\;\;\eta \leq 0\\ \Phi =3\eta \;\;\;for\;\;\;0<\eta \leq 0.333\\ \Phi =1\;\;\;for\;\;\;\eta >0.333 \end{array}\right..$$

The symbols used in Equations (3)–(15) denote the following: 〈 〉—Macaulay bracket, *σ_m_*—mean stress, *σ_i_*—effective stress, *σ*_1_—maximum principal stress, *τ_max_*—maximum shear stress, *η*—stress triaxiality, *ε*—effective strain, *A*—material constant (Hambli and Reszka [34] assumed that *A* = 0.424), *c*_1_ and *c*_2_—material constants.

## 3. Principle of the CWR Test

**Figure 1:**
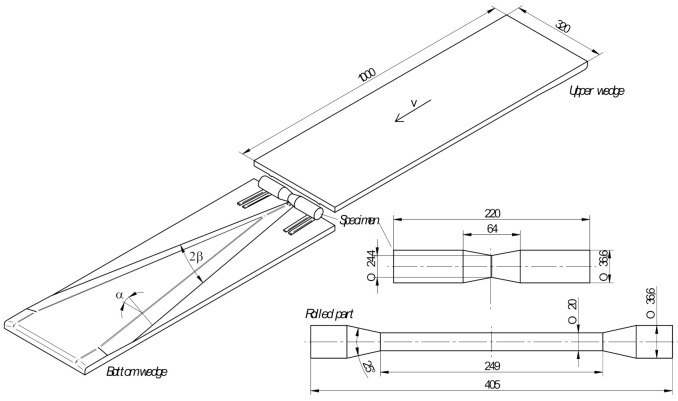
Schema of a new calibration test based on cross wedge rolling (CWR); dimensions in mm.

Designed to determine the critical damage in CWR, the new calibration test involves the use of wedge tools with variable geometry. Figure 1 shows the schema of the proposed test which is performed in the same way as the standard CWR process with the use of two flat wedge tools. One wedge tool (lower) is stationary, while the other (upper) moves linearly with a constant velocity *v*.

The billet for rolling is an axisymmetric specimen tapered in the centre. The change in the billet diameter *d*_0_ (with the rolled step diameter *d* maintained constant) leads to the change in deformation which is expressed via the reduction ratio *δ* defined as:

(16) $$\delta =\frac{d_{0}}{d}.$$

**Figure 2:**
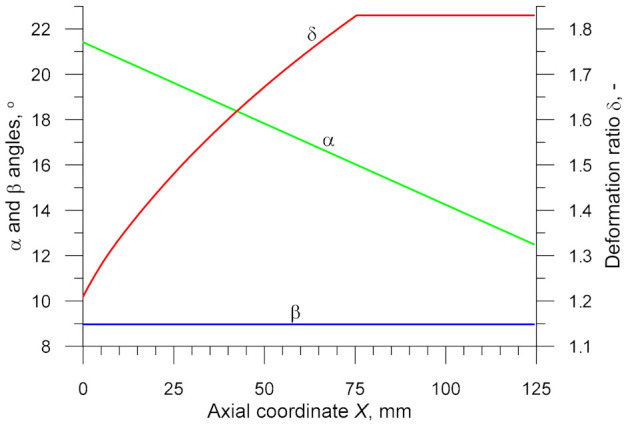
Parameters describing the formation of a specimen in the CWR test versus axial coordinate *X*.

The design of the wedge tool is based on the assumption that the wedge width increases at an angle of *β* = 9° over the entire tool length. The forming angle *α* (describing inclination of the wedge lateral face) is variable and linearly decreases from 21.4° to 12.5°. Figure 2 shows the basic parameters of the CWR process (*α*, *β*, *δ*) describing the formation of a step at a given distance *X* from the centre of the specimen (the axial coordinate *X* = 0 denotes plane symmetry).

According to the data in Figure 2, it can be seen that as the distance from the specimen’s centre increases, the forming process conditions gradually deteriorate and fracture becomes more probable (which results from an increase in *δ* and a reduction in *α*). At a certain point (depending on the material and the temperature *T*), the forming conditions become so unfavourable that the material begins to fracture.

To determine the critical damage *C_i_*, it is necessary to estimate experimentally the value of the axial coordinate *X_c_* where fracture will be initiated. After that, the calibration test must be modelled numerically in order to determine distributions of the damage function *f_i_* depending on the coordinate *X*. The critical damage *C_i_* will be the value of *f_i_* at the location denoted by *X_c_*.

A detailed description of the method for determining the critical damage *C_i_* of 42CrMo4 grade steel is given subsequently in the manuscript.

## 4. Experimental Tests

**Figure 3:**
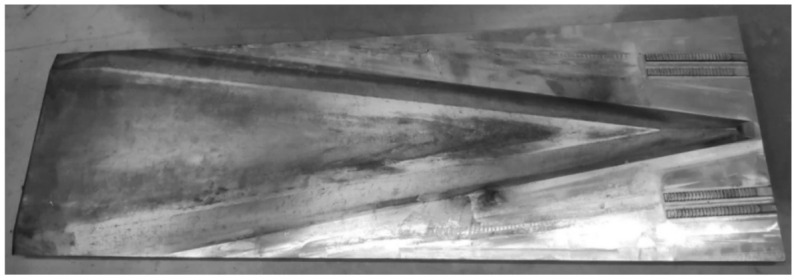
Tool (wedge with a variable forming angle α) used in the CWR test.

Experiments were conducted at the Lublin University of Technology, using a hydraulically driven flat-wedge rolling mill (SIGMA SA, Barak, Poland). The rolling mill was equipped with wedge tools (Figure 3), whose parameters were the same as those shown in Figure 1 and Figure 2.

**Figure 4:**
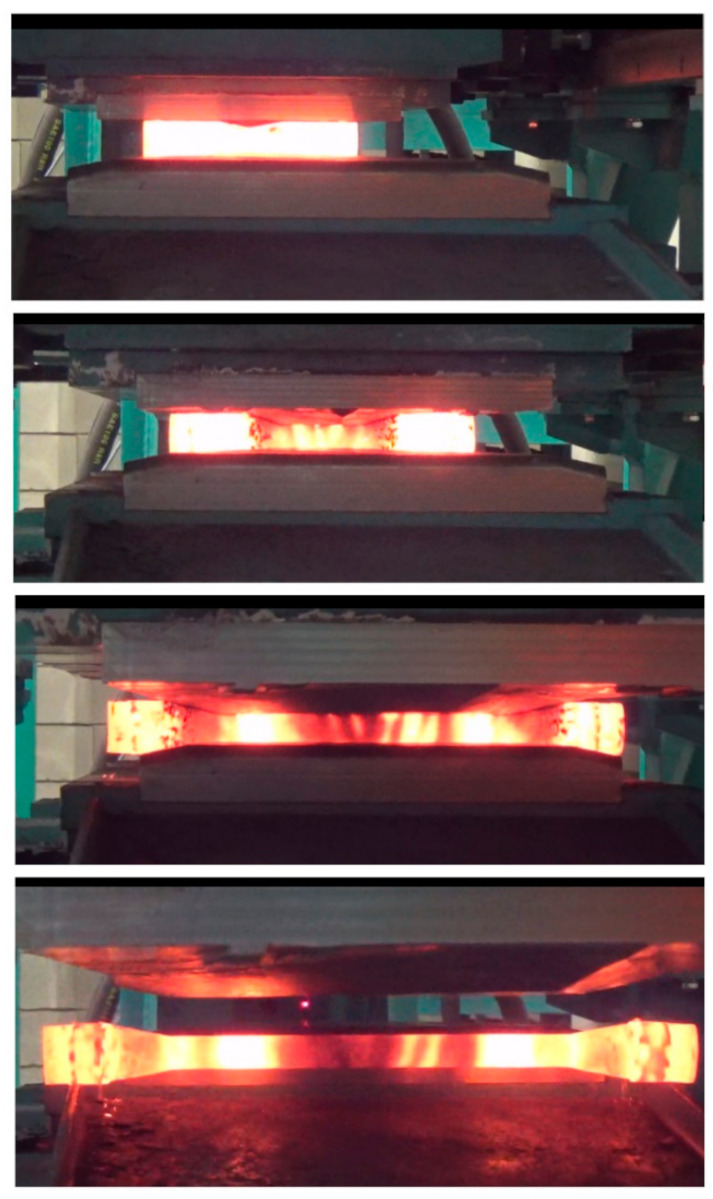
CWR tests conducted with a specimen preheated to 1000 °C.

Test specimens were prepared in compliance with Figure 1. They were made of 42CrMo4 grade steel. Prior to rolling, the specimens were preheated to different temperatures *T*_0_ (900 °C, 1000 °C and 1100 °C) in an electric chamber furnace (LAC s.r.o., Zidlochovice, Czech Republic). The preheated specimen was first placed on the lower tool using specifically designed guiding paths to ensure the correct position of the workpiece at an early stage of the rolling process. After that, the upper tool was set in motion; it was moved linearly with a velocity of *v* = 300 mm/s. During the rolling process, the specimen rolled over the lower (stationary) tool undergoes elongation. Figure 4 shows the CWR test conducted with the test specimen preheated to 1000 °C.

**Figure 5:**
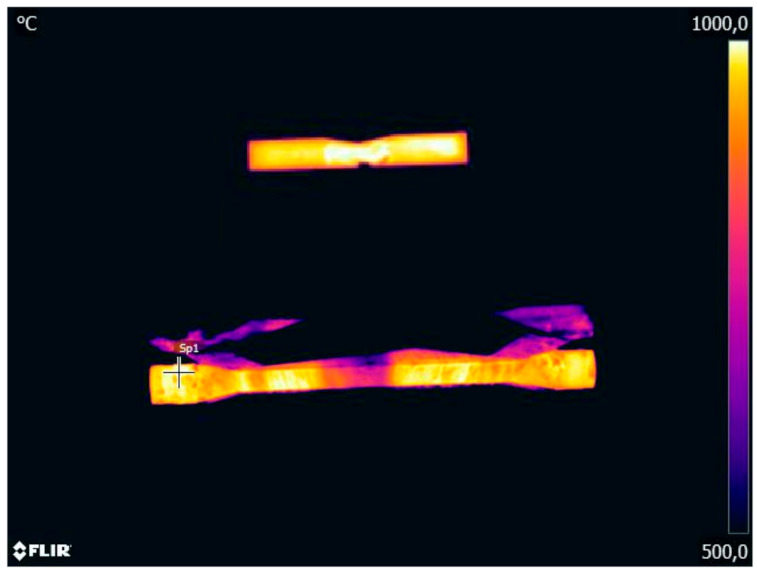
Temperature in the CWR test: (top) billet for rolling, (bottom) workpiece toward the end of the rolling process, conducted with the billet preheated to *T*_0_ = 1000 °C.

In the experiments, the force necessary to set into motion the movable wedge tool was measured. The results were then used to validate the numerical model of the CWR test. In addition, temperature was measured with an infrared camera (FLIR Systems, Inc., Winsonville, OR, USA). The results (Figure 5) demonstrate that the temperature on the surface of the specimen decreases during the rolling process. The highest decrease in temperature is observed in the specimen’s central zone that undergoes deformation at the beginning of the test.

**Figure 6:**
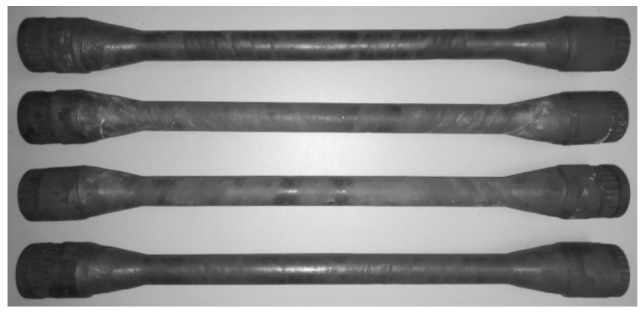
Examples of parts rolled in the CWR calibration test.

Figure 6 shows the examples of rolled parts obtained in the CWR test. Their shape and dimensions are as required (see Figure 1). On the surface of the rolled step one can observe the presence of spiral tracks that were formed due to contact of the workpiece with the tool edge.

**Figure 7:**
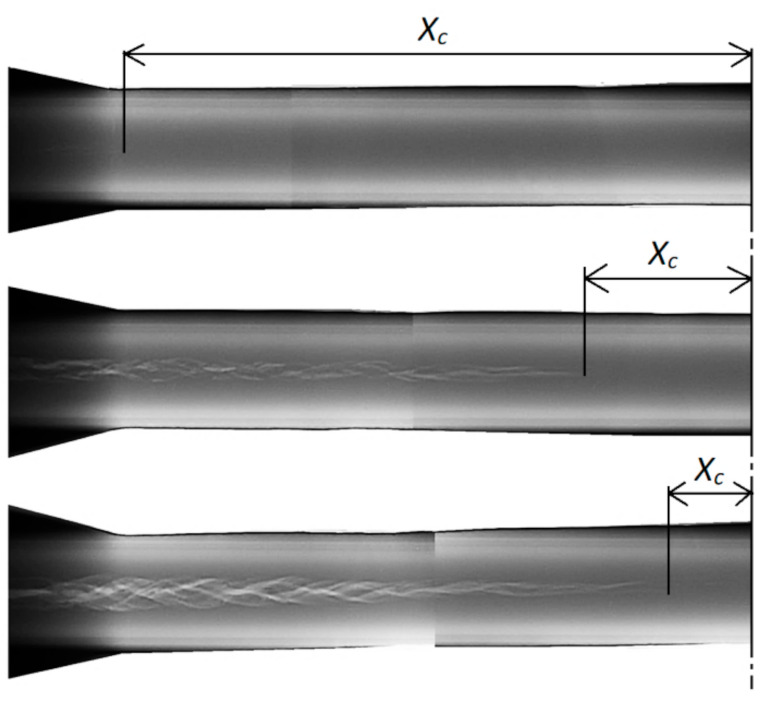
Radiograms of 42CrMo4 steel specimens rolled from billets preheated to (from top to bottom): 1100, 1000 and 900 °C.

Rolled parts were examined for internal fracture by radiography. Examples of radiograms obtained for parts rolled from billets preheated to different temperatures are shown in Figure 7. The results indicate the presence of internal cracks, their size depending on the temperature and axial coordinate *X*. The nature of these cracks points to the occurrence of considerable torsion in the axial zone of the material. Results obtained for three specimens preheated to the same temperature were used to estimate the average values of *X_c_* describing the location of fracture initiation. These average values are 16.7, 42.2 and 123.7 mm for the specimens preheated to *T*_0_ of 900, 1000 and 1100 °C, respectively. These average values of *X_c_* were then used to calculate the critical damage *C_i_*.

## 5. Numerical Analysis and Validation of the Numerical Model

Numerical simulations of the CWR test were performed in Simufact.Forming (v.15, MSC Software Company, Hamburg, Germany). This program was previously used to analyse numerous processes, such as the cross wedge rolling of shafts and axles [35,36,37], tube rolling [38,39,40], the helical rolling of balls [41,42,43], three-roll skew rolling [44,45,46] and cross rolling [47,48,49,50,51]. The numerical results of these studies showed high agreement with validating experimental data.

**Figure 8:**
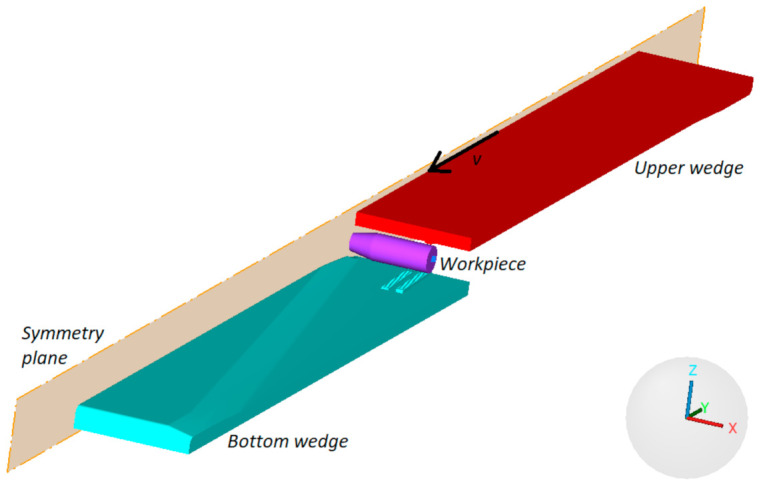
Geometric model of the CWR test designed using process symmetry.

Figure 8 shows the geometric model of the proposed CWR test, designed for the purpose of the numerical analysis. To reduce the computation time, process symmetry was employed and only half of the specimen was modelled. The wedge tools were modelled as ideally rigid bodies, whereas the specimen was assigned the properties of an elastic-plastic body. The velocity of the movable (upper) tool was set equal to 300 mm/s. The tools and specimens used in the numerical analysis had the same geometry as those used in the experiments, according to the specifications given in Figure 1 and Figure 2.

The material model of 42CrMo4 grade steel was obtained from the material database library of the Simufact.Forming software (v.15, MSC Software Company, Hamburg, Germany). This model is described with the following equation:

(17) $$\sigma_{F}=4628.8e^{-0.00345T}\varepsilon^{\left(-0.00000509T-0.03638\right)}e^{\left(-0.00000461T-0.01944\right)/\varepsilon }{\dot{\varepsilon }}^{\left(0.0001893T-0.04627\right)}$$

where *σ_F_* is the flow stress, MPa; *ε* is the effective strain, -; $\dot{\varepsilon }$ is the strain rate, s^−1^; *T* is the temperature, °C.

It was also assumed that friction would be described by the Tresca model:

(18) τ=m k,

where *τ* is the shear stress on contact surface, MPa; *m* is the friction factor (set equal to *m* = 0.85), -; *k* is the shear yield stress ($k=\sigma_{F}/\sqrt{3})$), MPa.

Three cases of the rolling process were analysed, each conducted with a different temperature of the billet, *T*_0_ = 900, 1000 and 1100 °C. The temperature of the tools during the rolling process was maintained constant at 50 °C. The exchange of heat between the tools and the material was described by the coefficient of heat exchange set equal to 10,000 W/m^2^K [19,21,45].

A model of the test specimen was meshed with 8 node hexahedral elements. It was assumed that all elements would have the same size of 1.5 mm. Remeshing was performed when the effective strain increased in any node by a value of 0.4.

**Figure 9:**
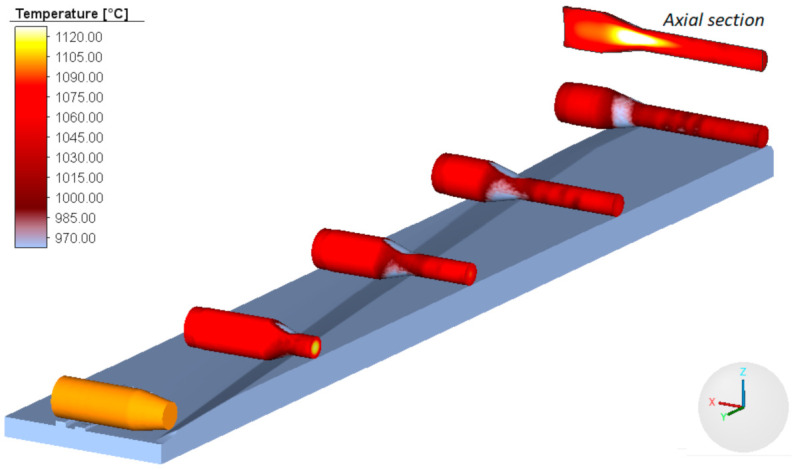
Successive stages of the CWR test conducted with the billet preheated to 1100 °C, with images showing the distribution of temperature.

Numerical results show agreement with the experimental results. All rolled parts have the required shape and the rolling process is free from any failure modes (e.g., uncontrolled slip, workpiece bending), which agrees with the experimental findings. Figure 9 shows one of the numerically modelled cases of the CWR process.

**Figure 10:**
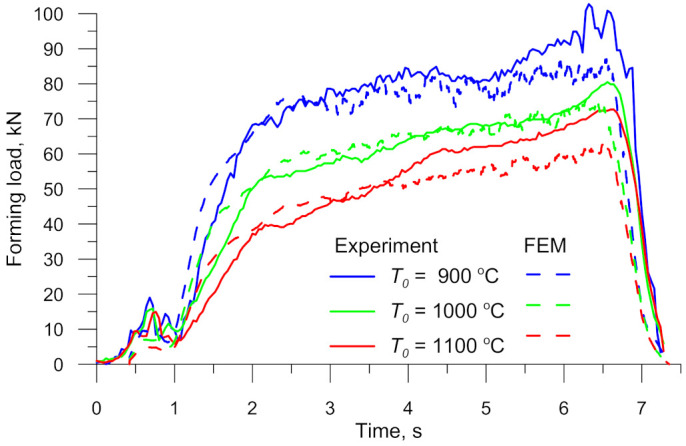
Experimental and FEM (Finite Element Method) forming loads in the CWR test versus billet temperature *T*_0_.

As previously mentioned, the force necessary for setting into motion the movable tool was measured in the experiments. The experimental findings are compared with the numerical results. The results plotted in Figure 10 demonstrate that the forming load gradually increases. The observed increase in the forming load results from both increasing the reduction ratio *δ* and decreasing the forming angle *α*, which leads to increasing the material-tool contact surface as the rolling process progresses. The numerical and experimental forces are in good agreement in qualitative terms.

Numerical and experimental forces necessary for specimen deformation in the analysed CWR process were quantitatively compared, based on the obtained force. The experimental forces were 138.95 kJ (for *T*_0_ = 900 °C), 109.81 kJ (for *T*_0_ = 1000 °C) and 94.35 kJ (for *T*_0_ = 1100 °C). On the other hand, the numerical obtained force values were slightly smaller, i.e., 129.83 kJ (for *T*_0_ = 900 °C), 106.95 kJ (for *T*_0_ = 1000 °C) and 86.13 kJ (for *T*_0_ = 1100 °C). The smaller numerical forces (by 6.56% for *T*_0_ = 900 °C, 1.71% for *T*_0_ = 1000 °C, 8.71% for *T*_0_ = 1100 °C), when compared to the experimental results, can be explained by the fact that during the real process there occurs additional resistance (e.g., friction on the rolling mill guides), which is not considered in the numerical simulation. Taking the above into account, it can be stated that the numerical model of the CWR test reflects the real process conditions very well.

## 6. Results and Discussion

**Figure 11:**
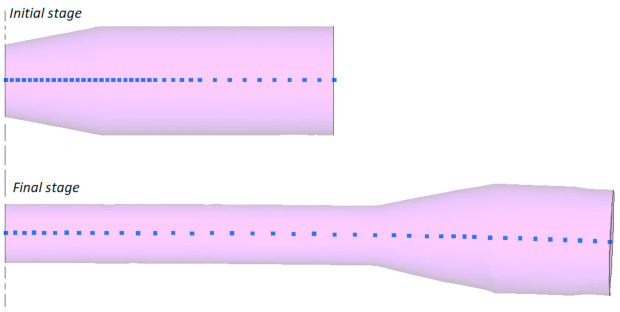
Location of virtual sensors used for registering stress and strain.

The critical damage in the center of the specimens was determined with the use of 40 virtual sensors. The arrangement and location of these sensors is shown in Figure 11. The sensors made it possible to register stresses and strains in the specimen, particularly in the region of a rolled cylindrical step. Stress and strain results captured with the virtual sensors were used to calculate the damage functions described by Equations (3)–(14). Calculations were made using Excel spreadsheets.

The state of stress in the centre of the specimen can be determined with the use of the stress triaxiality *η* and the Lode angle parameter Lode *θ*. The parameters *η* and *θ* have a considerable effect on fracture [52,53,54,55] and are connected with the stress invariants. The stress triaxiality *η* is defined as the ratio between the first stress invariant *σ_m_* and the second stress invariant *σ_i_*, hence

(19) $$\eta =\frac{\sigma_{m}}{\sigma_{i}}.$$

The Lode angle parameter *θ* depends on the second stress invariant *σ_i_* and on the third stress invariant *r*, and is expressed as:

(20) $$\theta =1-\frac{2}{\pi }\arccos\left[{\left(\frac{r}{\sigma_{i}}\right)}^{3}\right],$$

where

(21) $$\sigma_{m}=\frac{\sigma_{1}+\sigma_{2}+\sigma_{3}}{3},$$

(22) $$\sigma_{i}=\sqrt{\frac{1}{2}\left[{\left(\sigma_{1}-\sigma_{2}\right)}^{2}+{\left(\sigma_{2}-\sigma_{3}\right)}^{2}+{\left(\sigma_{1}-\sigma_{3}\right)}^{2}\right]},$$

(23) $$r={\left[\frac{27}{2}\left(\sigma_{1}-\sigma_{m}\right)\left(\sigma_{2}-\sigma_{m}\right)\left(\sigma_{3}-\sigma_{m}\right)\right]}^{\frac{1}{3}}$$

In the above equations, *σ*_1_, *σ*_2_, *σ*_3_ denote the principal stresses.

The parameters *η* and *θ* vary during the rolling process. Therefore, for comparative purposes, their average values were determined, depending on the variations in effective strain recorded with the sensors. This was done using the following equations:

(24) $$\eta_{av}=\frac{1}{\varepsilon }\int_{0}^{\varepsilon }\eta \;d\varepsilon ,$$

(25) $$\theta_{av}=\frac{1}{\varepsilon }\int_{0}^{\varepsilon }\theta \;d\varepsilon .$$

**Figure 12:**
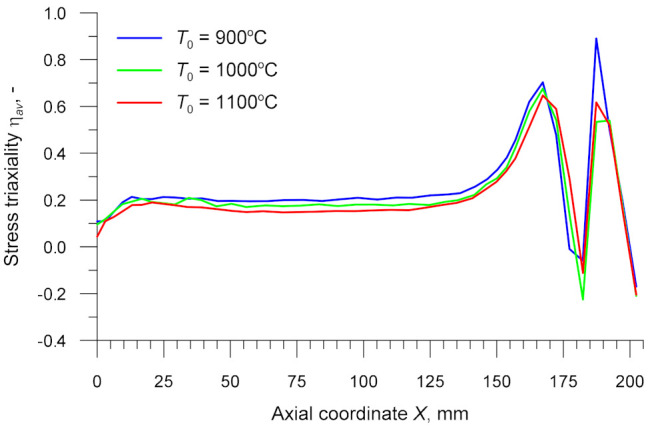
Stress triaxiality in the axial zone of the specimen versus billet temperature *T*_0_.

Figure 12 shows the stress triaxiality *η_av_* in the axial zone of the specimen. The focus was put on the variations in this parameter occurring in the cylindrical part of the specimen, i.e., when *X* < 124.5 mm. An analysis of the data in Figure 12 reveals that *η_av_* is relatively constant over the reduced section, ranging from 0.17 to 0.21 (the exception is the specimen’s centre where the value of *η_av_* is slightly lower). The temperature *T*_0_ has practically no effect on the value of *η_av_*. According to the results reported in [55,56], the mechanism of fracture depends on the stress triaxiality. When *η* ≥ 0.333, material fracture is caused by void nucleation, growth and coalescence, when *η* ≤ 0, it is caused by shear, whereas at 0 < *η* < 0.333 material fracture may be caused by both mechanisms. The latter case can be observed in the CWR test.

**Figure 13:**
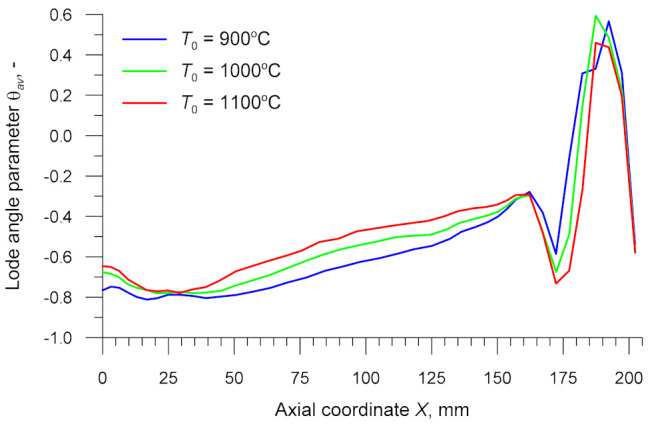
Lode angle parameter in the axial zone of the specimen versus billet temperature *T*_0_.

The results demonstrate that a change in the rolling parameters leads to a change in the Lode angle parameter *θ_av_* (Figure 13). The value of this parameter ranges from −0.81 to −0.43 in the cylindrical section of the specimen. The increase in *θ_av_* results both from a decrease in the forming angle *α* that occurs with increasing the coordinate *X* and from an increase in the forming temperature *T*. The increase in *θ_av_* means that the effect of shear stresses on the forming process has increased.

**Figure 14:**
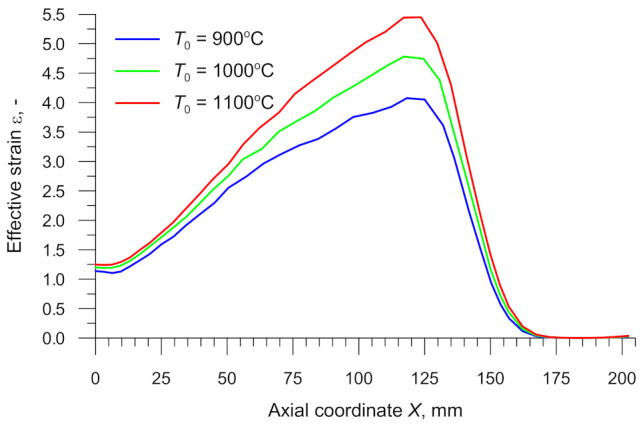
Effective strain in the axial zone of the specimen versus billet temperature *T*_0_.

Figure 14 shows the effective strains in the axial zone of the specimens rolled from billets preheated to different temperatures. It can clearly be observed that the strain increases with the distance from the plane of symmetry (denoted by *X* = 0 mm). This is a combined effect of increasing the billet diameter *d*_0_ and decreasing the forming angle *α*, resulting in a higher number of deformation cycles (the number of revolutions of the workpiece) that are necessary for reducing the specimen’s diameter to the required value *d*. The highest effective strains are located in the region where the cylindrical part of the specimen changes into tapered (i.e., when *X* ≈ 124.5 mm). An analysis of the effective strains obtained for different billet temperatures shows that the effective strains increase with increasing the temperature. Given the identical shape and dimensions of the specimens, this effect can only be explained by a higher rate of tangential flow of the material that causes changes in non-dilatational strains.

An important aspect of the CWR test concerns rapid variations in the temperature of the specimen. The heat of the specimen is carried away to the colder tools, which leads to a decrease in the temperature on the specimen surface (see Figure 5 and Figure 9). The highest temperature decrease can be observed in the specimen’s centre that undergoes deformation at the beginning of the test. In addition to this, it can be observed that the axial zone of the specimen undergoes intensive deformation. In total, 90% of the deformation work is exchanged into heat, which leads to an increase in the temperature of the material. This effect can be observed in Figure 9 showing the axial section of the specimen. In the region where the specimen changes from cylindrical into tapered (which occurs toward the end of the test), the temperature of the material is higher than the billet temperature. Therefore, it is important to calculate the average temperature *T_av_* in the centre of the specimen. This temperature is determined using the following formula:

(26) $$T_{av}=\frac{1}{\varepsilon }\int_{0}^{\varepsilon }T\;d\varepsilon .$$

**Figure 15:**
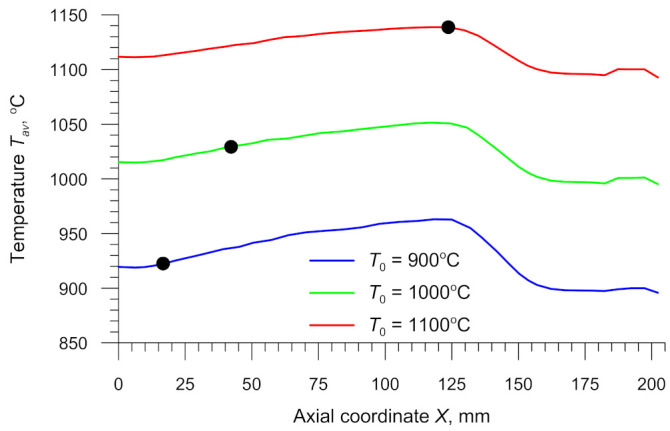
Average temperature in the axial zone of the specimen versus billet temperature *T*_0_; the symbol “●” marks the location of fracture initiation.

Results obtained for the three analysed cases of the CWR process are shown in Figure 15. It can be observed that during the forming process the average temperature *T_av_* is higher than the billet temperature *T*_0_. The temperature increases with the distance from the centre of the specimen, which results from the fact that the deformation work is more intensive and thus more heat is generated. The temperature *T_av_* decreases in the tapered section of the specimen (*X* > 124.5 mm), where the effective strain decreases rapidly (see Figure 14).

Based on the data in Figure 15, one can determine the temperature of the material at the location of fracture initiation. These loci were identified via experimental tests and are marked in the plot with the symbol “●”. The results demonstrate that, for the specimen rolled from the billet preheated to *T*_0_ = 900 °C, this temperature is 924.8 °C. For the specimen rolled from the billet preheated to *T*_0_ = 1000 °C, this temperature is 1029.3 °C, and for that preheated to *T*_0_ = 1100 °C it is as high as 1138.7 °C. These temperatures were then used to determine the relationship between the critical damage *C_i_* and the temperature *T*.

**Figure 16:**
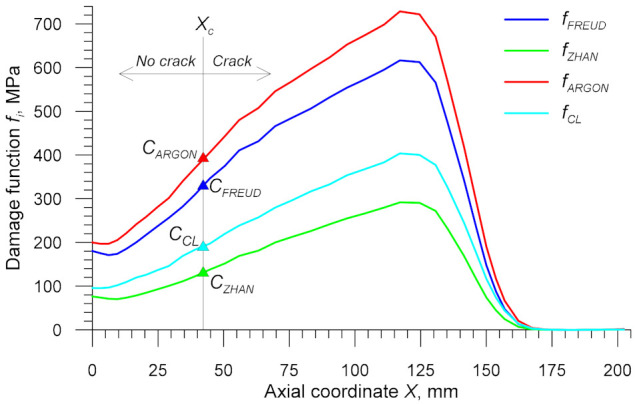
Stress-based damage functions in the axial zone of the specimen in the CWR test, with *T*_0_ = 1000 °C (the axial coordinate *X* = 0 mm denotes symmetry plane).

**Figure 17:**
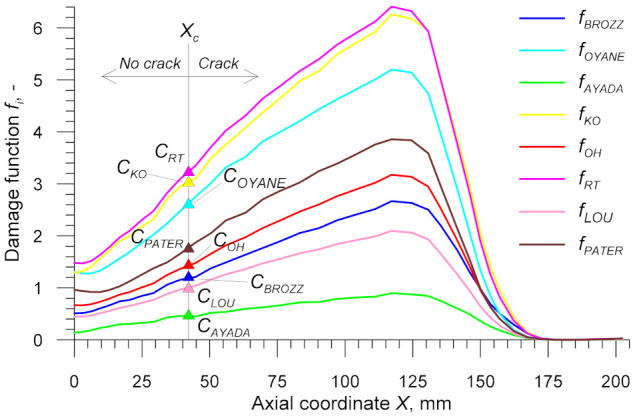
Dimensionless damage functions in the axial zone of the specimen in the CWR test, with *T*_0_ = 1000 °C (the axial coordinate *X* = 0 mm denotes symmetry plane).

Using both data captured by the virtual sensors and Equations (3)–(15), it was possible to determine distributions of the damage function in the centre of the specimens. The results obtained for the specimens rolled from the billet preheated to *T*_0_ = 1000 °C are shown in Figure 16 and Figure 17 for the stress-based and dimensionless damage functions, respectively. The behaviour pattern of all plotted damage functions is similar to that of effective strain shown in Figure 14. With increasing the distance from the centre of the specimen (i.e., from *X* = 0 mm), the damage function *f_i_* increases in the cylindrical section of the specimen (*X* ≤ 124.5 mm), which makes it possible to determine the critical damage *C_i_*. This is done by calculating the value of *f_i_* at a distance *X_c_* from the specimen centre, as shown in Figure 16 and Figure 17. Obtained critical damage values are given in Table 1. These values depend on the temperature *T_av_* observed at fracture location and they clearly increase with the temperature. This proves that the temperature plays a key role in material fracture in the CWR process.

Given the strong relationship between the critical damage *C_i_* and the temperature *T* in CWR, it is possible to use the following mathematical formula:

(27) $$C_{i}=eT^{2}+fT+g,$$

where *e*, *f*, *g* are the equation parameters according to Table 2.

Equation (27) can be implemented to FEM-based program for CWR analysis and used to determine the critical damage *C_i_* in the nodes (virtual sensors) for a given time step. A comparison of the value of *C_i_* and the value of *f_i_* helps determine the initiation of internal fracture, which occurs when $f_{i}\geq C_{i}$.

**Figure 18:**
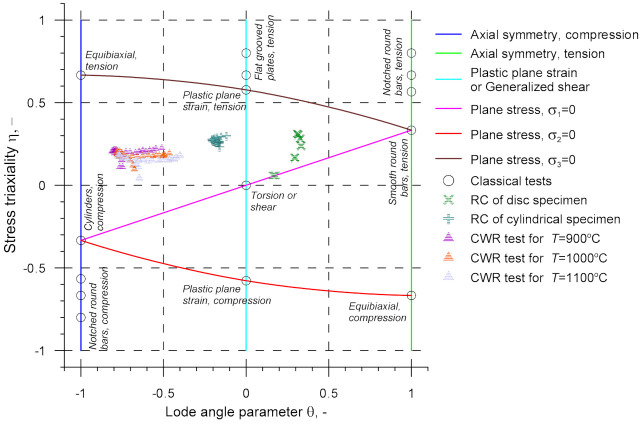
Stress triaxiality in the CWR test and other tests used for damage function calibration.

It is also important that the proposed CWR test be compared with previous calibration tests. To this end, one should use the plot made in the *η*-*θ* plane (Figure 18) showing the stresses in the CWR test, rotary compression tests [17,18], and classical tests based on tension, compression and torsion [57,58]. The stress in the CWR test was measured with 25 virtual sensors located in the cylindrical section of the specimen. An analysis of the data in Figure 18 reveals that the stress triaxiality *η* in the CWR test is the same as that obtained in the rotary compression tests. At the same time, the Lode angle parameter *θ* is much lower. This proves definitively that the stress occurring in the CWR test differs from that induced in previous calibration tests.

Last but not least, it should be mentioned that the proposed CWR test has some limitations. As can be observed in Figure 7, the crack in the specimen rolled from the billet preheated to *T*_0_ = 1100 °C is hardly detectable. Hence, any further increase in the material workability (e.g., due to an increase in the temperature *T*) will mean that fracture will not occur. Therefore, future research is needed to overcome this limitation, e.g., by designing new shapes of wedge tools or test specimens.

## 7. Conclusions

The results of this study lead to the following conclusions:

- The phenomenological criteria can be used to analyse material fracture in CWR processes provided that one uses the critical damage values determined in a calibration test in which the modelled stress reflects the real stress;
- The critical damage can be best determined with the new CWR-based calibration test which uses wedge tools with a variable forming angle and diameter-variable specimens; any change in these parameters causes (with an increase in the distance from the specimen centre) deterioration in the forming conditions and is thus conducive to material fracture;
- Despite a relatively long forming time in the CWR test, the temperature in the centre of the specimen increases (which results from the exchange of deformation work into heat);
- The use of the new CWR test and 12 criteria of ductile fracture made it possible to determine the critical damage of 42CrMo4 steel specimens formed in the temperature range of 900–1100 °C;
- The critical damage of 42CrMo4 steel depends to a great extent on the forming temperature; the critical damage increases with the forming temperature.

## Figures and Tables

**Table 1:** Critical damage of 42CrMo4 grade steel depending on the forming temperature *T* (assuming that *T* = *T_av_*), as determined in the CWR test.

*C_i_*	*T* = 924.8 °C	*T* = 1029.3 °C	*T* = 1138.7 °C
*C_FREUD_*	272.0 MPa	329.8 MPa	533.6 MPa
*C_BROZ_*	0.729	1.184	2.943
*C_OYANE_*	1.564	2.628	5.837
*C_AYADA_*	0.296	0.455	0.915
*C_ZHAN_*	103.7 MPa	130.9 MPa	261.7 MPa
*C_ARGON_*	326.8 MPa	389.8 MPa	620.1 MPa
*C_CL_*	158.6 MPa	191.0 MPa	348.3 MPa
*C_KO_*	1.892	3.034	6.841
*C_OH_*	0.853	1.427	3.587
*C_RT_*	1.969	3.233	7.054
*C_LOU_*	0.609	1.006	2.306
*C_PATER_*	1.026	1.774	4.523

**Table 2:** Parameters of Equation (27) describing the relationship between the critical damage *C*_i_ and the forming temperature *T*.

*C_i_*	*e*	*f*	*g*
*C_FREUD_*	0.006117	−11.398	5581.45
*C_BROZ_*	0.00005467	−0.10244	48.711
*C_OYANE_*	0.00008959	−0.16490	77.43
*C_AYADA_*	0.00001256	−0.02303	10.850
*C_ZHAN_*	0.004354	−8.2454	4004.26
*C_ARGON_*	0.007008	−13.089	6337.21
*C_CL_*	0.005245	−9.9357	4860,03
*C_KO_*	0.0001115	−0.20694	97.905
*C_OH_*	0.00006647	−0.12436	59.013
*C_RT_*	0.0001068	−0.19662	92.465
*C_LOU_*	0.00003777	−0.07000	33.045
*C_PATER_*	0.00008384	−0.15663	74.174

## Data Availability

Data is contained within the article.
